# Update on therapeutic temperature management

**DOI:** 10.1186/cc11259

**Published:** 2012-06-07

**Authors:** Gregor Broessner, Marlene Fischer, Gerrit Schubert, Bernhard Metzler, Erich Schmutzhard

**Affiliations:** 1 Department of Neurology, Medical University, Innsbruck, Austria; 2 Department of Neurosurgery, Medical University, Innsbruck, Austria; 3 Department of Cardiology, Medical University, Innsbruck, Austria

## 

It is a pleasure to announce the 2nd Innsbruck Hypothermia Symposium. We are very happy that *Critical Care *has agreed to publish extended abstracts submitted by invited renowned scientists from all over the world; that is, Europe, the Americas, Asia. Neuroprotection - potentially achieved by targeted temperature management (that is, therapeutic hypothermia or prophylactic controlled normothermia) - is essential in emergency and acute care management of various severe neurologic and cardiologic diseases. Beyond neuroprotection - for this aim, therapeutic hypothermia has been established after resuscitation of patients with cardiac arrest due to a shockable arrhythmia and in neonatal asphyxic encephalopathy - therapeutic hypothermia and prophylactic controlled normothermia have been published in single case reports, retrospective, open, but also in prospective randomised controlled trials in many other emergency disciplines in which both neuroprotection and protection of other organs and tissues are the target of our therapeutic endeavours. The Medical University Innsbruck, Austria, is happy to organise this conference on temperature management, therapeutic hypothermia and prophylactic normothermia respectively, to be held in Portoroz, Slovenia. In accordance with the first Meeting on Hypothermia, which was held in Miami, Florida, USA (CHilling At the Beach), we are proud to suggest the acronym CHAB standing for take Care for Heart And Brain, characterising the major target organs of therapeutic and, possibly also, prophylactic temperature management. Again, we have been able to gather most renowned scientists, neurointensivists and intensivists, emergency physicians, cardiologists and other specialists to cover the entire scientific and clinical spectrum of emergency temperature management, technical aspects of cooling and management of potential complications including shivering, but also temperature management in neurology, neurosurgery, intensive care medicine, in the operation theatre, cardiology, infectious diseases, and so forth. Beyond that we cross borders and discuss hypothermia and intracranial pressure, pharmacodynamics in hypothermic patients and the influence of hypothermia onto pharmacokinetics/pharmacodynamics, hypothermia in refractory status epilepticus or heat stroke, hypothermia and advanced neuromonitoring, hypothermia and nutrition, shivering and the critical issue of rewarming, amongst other topics.

The aim of this symposium is to enhance the knowledge on temperature management, increase the readiness and stimulate the preparedness to institute therapeutic hypothermia and/or prophylactic controlled normothermia, respectively, in patients in need of tissue and organ protection, uncontrolled body temperatures possibly adding - *per se *- to neuronal damage. Knowing the medical literature and knowing the issue of potentially life-threatening side effects and complications incurred by this invasive therapeutic manoeuvre, it is the foremost aim of this symposium and this supplementary issue of *Critical Care *to discuss all these aspects of targeted temperature management in emergency, critical care and, in particular, neurocritical patients and conditions. For this reason the organisers have agreed that the discussion of these various issues, being so important for general critical care, neurocritical care and emergency medicine, must be distributed as widely as possible, making it available to critical care and neurocritical care specialists all over the world. Therefore we are extremely grateful to the Editors of *Critical Care *for providing a forum for all of the extended abstracts of all invited speakers, covering the entire field of adult emergency and critical care medicine. We do hope and we are convinced that this supplementary issue will be a source of inspiration and knowledge, hopefully becoming a work of reference for intensivists, neurologists, neurointensivists, cardiologists and all emergency physicians alike. It is the aim of the organisers to establish a series of such symposia within the next years in order to keep up with all the developments in this field and to maintain the highest possible level of knowledge of targeted temperature management in the community of emergency and intensive care physicians.

